# Mini-review: research and progress of oxeiptosis in diseases

**DOI:** 10.3389/fcell.2024.1428250

**Published:** 2024-06-20

**Authors:** Ke-Qian Chen, Shu-Zhi Wang, Hai-Bo Lei, Xiang Liu

**Affiliations:** ^1^ Department of Clinical Pharmacy, Xiangtan Central Hospital, Xiangtan, China; ^2^ Institute of Pharmacy and Pharmacology, School of Pharmaceutical Sciences, Hengyang Medical School, University of South China, Hengyang, China

**Keywords:** oxeiptosis, ROS, KEAP1, PGAM5, AIFM1

## Abstract

Oxeiptosis is a novel cell death pathway that was introduced in 2018. As a form of regulated cell death, it operates independently of caspases and is induced by ROS. Distinguished from other cell death pathways such as apoptosis, necroptosis, pyroptosis, and ferroptosis, oxeiptosis features unique damage causes pivotal genes, and signaling pathways (KEAP1/PGAM5/AIFM1). Emerging studies indicate that oxeiptosis plays a significant role in the progression of various diseases and its regulation could serve as a promising therapeutic target. However, the precise molecular mechanisms underlying oxeiptosis remain to be fully elucidated. In this mini-review, we systematically summarize the latest developments in oxeiptosis-related diseases while detailing the molecular mechanisms and regulatory networks of oxeiptosis. These insights offer a foundation for a deeper understanding of oxeiptosis.

## 1 Introduction

Reactive oxygen species (ROS) are by-products of biological aerobic metabolism, encompassing a variety of entities such as superoxide, hydroxyl radical, singlet oxygen, peroxide, ozone, and other radicals and non-radical species (Halliwell, 2024). Research underscores the pivotal role of ROS in cellular signaling (Thannickal and Fanburg, 2000). Several triggers including viral infections, allergies, inflammatory cytokines, allograft rejection, ultraviolet exposure, and heat can elevate intracellular ROS levels, contributing to the advancement of numerous pathological conditions such as carcinogenesis, neurodegeneration, atherosclerosis, diabetes, and aging (Pan et al., 2022). In 2018, Holze et al., 2018 found that high concentrations of O_3_ or H_2_O_2_ can induce oxeiptosis. Mouse airway cells exposed to ozone and HeLa cells infected by influenza A virus instigate exogenous oxidative stress. Oxeiptosis is a caspase-independent and ROS-induced cell death pathway. Holze et al., 2018 also found mouse embryonic fibroblasts showed lower viability and apoptotic membrane blebbing after treatment with H_2_O_2_. Meanwhile, Caspase inhibitor has not changed the induction of cell death. Similarly, inhibitors of ferroptosis, apoptosis, and necroptosis did not alter the H_2_O_2_-induced toxicity (Holze et al., 2018). Beyond ROS, triggers like viral infection and 5-Fluorouracil attached to magnetic nanoparticles can also induce oxeiptosis (Dabaghi et al., 2020). Oxeiptosis is characterized by the activation of the Kelch-like ECH-associated protein 1 (KEAP1)/phosphoglycerate mutase 5 (PGAM5)/apoptosis-inducing factor mitochondria-associated 1 (AIFM1) signaling pathway. Current research indicates that oxeiptosis is connected to various biological functions. For example, depletion of PGAM5 and AIFM1 leads to neurological dysfunctions in mice (Liang et al., 2021). Moreover, mutations in KEAP1 are associated with lung and prostate cancers (Zhang et al., 2010). Thus, a deeper understanding of the mechanisms underlying oxeiptosis and its implications in disease could prove highly significant for future research endeavors.

## 2 Signaling pathway of oxeiptosis

**FIGURE 1:**
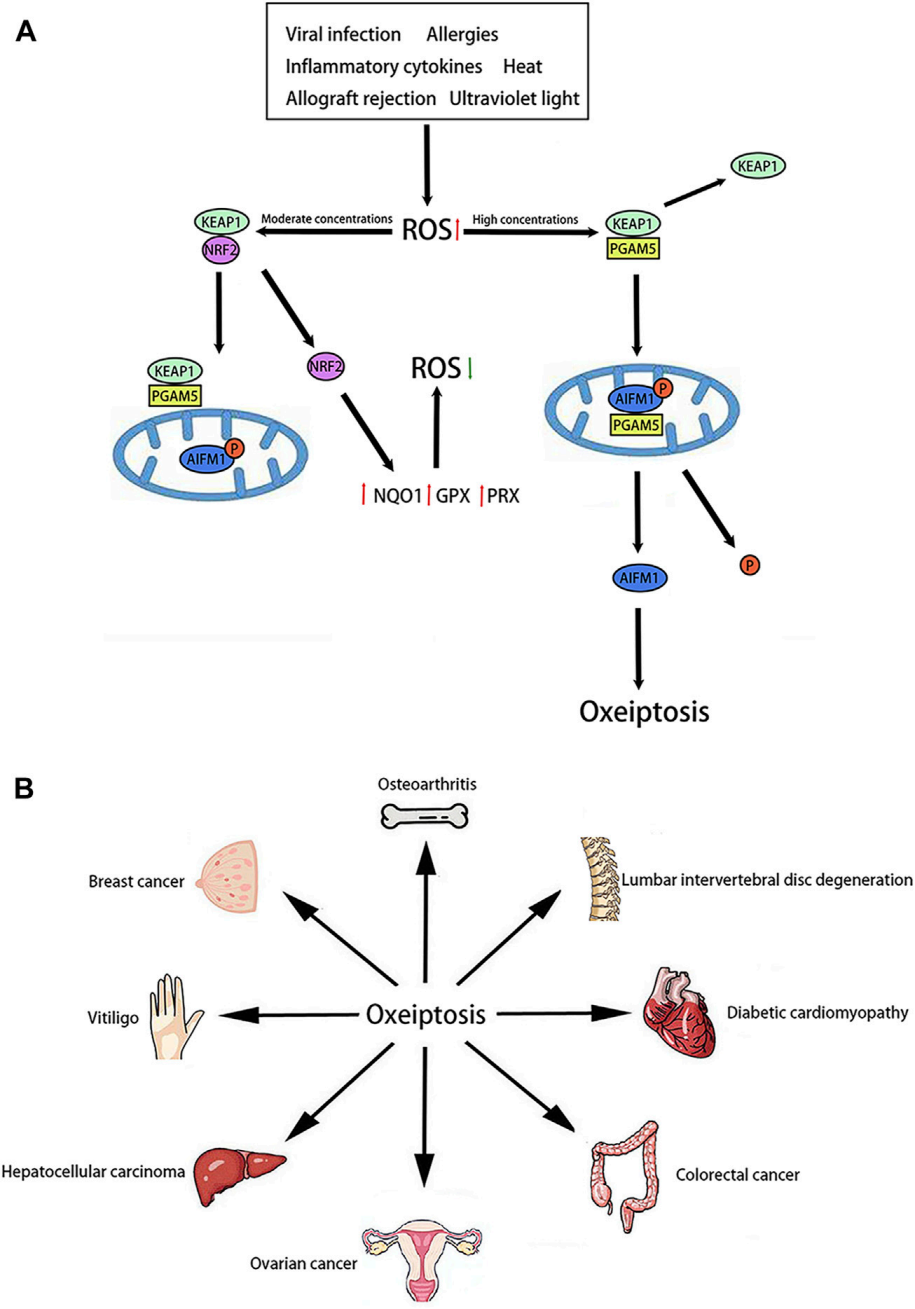
Molecular pathway and role of oxeiptosis **(A)**: Molecular pathway of oxeiptosis is characterized by the activation of the KEAP1/PGAM5/AIFM1. **(B)**: Oxeiptosis plays an important role in various organs, such as the bone, spine, heart, colon, ovary, liver, skin, breast and so on.

KEAP1 is a well-studied sensor capable of measuring ROS levels (Holze et al., 2018). Research points out that KEAP1 consists of five distinct domains: 1) the N-terminal region, 2) the broad complex, tramtrack and bric-a-brac domain, 3) the intervening region, 4) the double glycine repeats or Kelch domain, 5) the C-terminal region (Abed et al., 2015). As an endogenous inhibitor of nuclear factor erythroid 2-related factor 2 (NRF2), KEAP1 facilitates the continuous degradation of NRF2 via 27 cysteine residues located in its C-terminus (Tian et al., 2022). The KEAP1/NRF2 pathway is renowned for its protective role against oxidative stress. Under steady state conditions, the transcription factor NRF2 is retained within the cytosol by KEAP1. Under moderate ROS concentrations, NRF2 is dissociation form KEAP1. More and more NRF2 enters the nucleus. Meanwhile, NRF2 is capable of activating antioxidant response element (ARE) and further activating some downstream genes such as Heme oxygenase-1(HO-1), NADPH quinone oxidoreductase 1(NQO1), glutathione peroxidase (GPX) and peroxidase (PRX) (Park et al., 2023). Under high ROS concentrations, KEAP1 can mediate oxeiptosis through an NRF2-independent pathway (Park et al., 2023). This process is characterized by the activation of the KEAP1/PGAM5/AIFM1 pathway (Figure 1A). PGAM5 is a new regulator of mitochondrial homeostasis. PGAM5 activates mitochondrial biogenesis and mitophagy to promote a cellular compensatory response when mitochondria are mildly damaged, whereas severe damage to mitochondria leads to PGAM5 inducing excessive mitochondria fission, which eventually evoke cell death (Liang et al., 2021). As an interaction partner of KEAP1, PGAM5 is also a key downstream effector in oxeiptosis. When ROS concentrations are high, PGAM5 dissociates from KEAP1 and migrates to the mitochondria (Scaturro and Pichlmair, 2018). AIFM1 is a gene located on the X chromosome, coding for Apoptosis-Inducing Factor (AIF), a mitochondrial flavoprotein involved in caspase-independent cell death (Diodato et al., 2016). Inside the mitochondria, PGAM5 dephosphorylates the Ser116 residues of AIFM1, finally leading to oxeiptosis (Scaturro and Pichlmair, 2018). Notably, the interaction between PGAM5 and AIFM1 diminishes when cells undergo treatment with moderate ROS concentrations, but can be restored with the application of a ROS scavenger (Scaturro and Pichlmair, 2019).

## 3 Difference between oxeiptosis and other cell death pathways

Recent studies have shown that oxeiptosis operates in parallel with other cell death pathways and leads to a non-inflammatory, caspase-independent, apoptosis-like cell death phenotype. Therefore, it is crucial to differentiate oxeiptosis from other cell death pathways. Oxeiptosis is a ROS-induced cell death pathway. However, other cell death pathways such as apoptosis, necroptosis and ferroptosis are also triggered by ROS (Mbaveng et al., 2021). Research indicates that ferroptosis requires Fe^2+^ to participate in ROS production, while oxeiptosis does not (Li et al., 2020). Additionally, ROS generation in apoptosis and necroptosis involves the RIPK1/RIPK3 pathway and the Bcl2/Bax/Caspase-3 pathway, respectively (Bertheloot et al., 2021). Oxeiptosis, however, relies on the KEAP1/PGAM5/AIFM1 pathway. These findings suggest that ROS accomplish different death ways by selecting different pathways, although the reasons for pathway selection remains unclear. Morphologically, oxeiptosis exhibits no distinctive characteristics and resembles apoptosis (Holze et al., 2018). Apoptosis is characterized by cellular volume reduction, formation of apoptotic bodies, and cellular fragmentation (Ketelut-Carneiro and Fitzgerald, 2022). Ferroptosis is characterized by mitochondrial shrinkage and rupture of the mitochondrial outer membrane (Ketelut-Carneiro and Fitzgerald, 2022). Necroptosis is characterized by the destruction of cell membranes and the swelling of cells and organelles (Ketelut-Carneiro and Fitzgerald, 2022). These morphological characteristics facilitate the differentiation of oxeiptosis. In addition, the factors that induce oxeiptosis and other cell death pathways, the presence or absence of inflammation, and the associated inhibitors are also different.

## 4 Oxeiptosis and diseases

Recent studies suggest that oxeiptosis may be involved in the development of various diseases (Figure 1B). Initially, oxeiptosis appears to be linked to the occurrence and development of inflammation. In models of ozone exposure and viral infection, pgam5^−/−^mice exhibited severe inflammation and tissue damage (Tang et al., 2019). These findings suggest that oxeiptosis may inhibit inflammation. Moreover, oxeiptosis is implicated in the progression of osteoarthritis. Activation of the KEAP1/NRF2 signaling pathway attenuated osteoarthritis by reducing the expression of inflammatory factors (Fan et al., 2024).

Backache is a leading cause of movement disorders and disability. Research indicates that lumbar intervertebral disc degeneration is identified as a potential cause of backache (Chiu et al., 2024). Within the intervertebral discs, mitochondrial damage in nucleus pulposus cells leads to an increase in intracellular ROS, exacerbating lumbar intervertebral disc degeneration (Chiu et al., 2024). Chen X et al. discovered that oxeiptosis is triggered in nucleus pulposus cells (Chen et al., 2024). Inhibiting the oxeiptosis could slow the progression of lumbar intervertebral disc degeneration. Notably, CircFOXO3, a specific circRNA, regulates oxeiptosis in nucleus pulposus cells through the miR-185-3p/miR-939-5p/ASIC1 signaling pathway (Chen et al., 2024).

Vitiligo is an autoimmune skin disease caused by the destruction of epidermal melanocytes (Ezzedine et al., 2015). Unfortunately, current treatments for vitiligo are not very effective. Previous studies have highlighted oxidative stress as a significant factor in the destruction of melanocytes during vitiligo pathogenesis (Chang and Ko, 2023). Elevated levels of ROS can lead to molecular and organelle dysfunction, ultimately resulting in melanocyte death (Xuan et al., 2022). Chen J et al. observed oxeiptosis in vitiligo patients (Chen et al., 2021). ROS‐treated melanocytes exhibited oxeiptosis (Chen et al., 2021). Kang et al., 2022 reported that KEAP1 and PGAM5 were upregulated in H_2_O_2_-treated melanocytes, and AIFM1 was dephosphorylated at Ser116 (Kang et al., 2022). These findings indicate that the KEAP1/PGAM5/AIFM1 signaling pathway is activated in melanocytes.

Psoriasis is a papulosquamous skin disease characterized by the hyperproliferation of keratinocytes (Boehncke and Schön, 2015). The pathogenesis of this condition involves the production and accumulation of ROS, which can lead to abnormal differentiation and proliferation of keratinocytes, subsequently activating aberrant immune responses and inflammation (Barygina et al., 2019). Consequently, inhibiting the production of ROS is an effective strategy for alleviating psoriasis. You et al., 2023 found that KEAP1 and p-AIFM are highly expressed in psoriasis lesions. Simultaneously, the knockdown of KEAP1 can induce oxeiptosis. Additionally, 4-octylitaconate, an agonist of oxeiptosis, helps alleviate psoriasis by inhibiting KEAP1 (You et al., 2023).

Oxeiptosis also holds potential as a mechanism for treating hepatocellular carcinoma. Lin et al., 2023 screened a total of 69 core oxeiptosis genes, including members of the T-complex polypeptide1 family, Dead-box family, and heterogeneous nuclear ribonucleoprotein family. These genes have been identified as prognostic risk factors for hepatocellular carcinoma (Li et al., 2008). Phenethyl isothiocyanate (PEITC) and dasatinib have been shown to inhibit the growth of hepatocellular carcinoma by activating oxeiptosis (Strusi et al., 2023).

Furthermore, several studies have suggested that oxeiptosis may play a role in diabetic endothelial dysfunction and diabetic cardiomyopathy (Chen et al., 2020; Shen et al., 2023). Since excessive ROS production is a vital factor during diabetic endothelial dysfunction and diabetic cardiomyopathy, oxeiptosis maybe a contributor to diabetic endothelial dysfunction and diabetic cardiomyopathy.

## 5 Regulator of oxeiptosis

OTUD1 plays a crucial role in regulating inflammatory and oxidative stress responses (Oikawa et al., 2023). Previous studies have found that OTUD1 stabilizes the p53 tumor suppressor as a biomarker of thyroid cancer. OTUD1 also upregulates the expression of p21 and Mdm2, thus accelerating apoptosis (Piao et al., 2017). Oikawa D et al. demonstrated through mass spectrometric analysis that OTUD1 can bind to KEAP1 via an ETGE motif (Oikawa et al., 2022). In Otud1^−/−^mice, both inflammation and oxeiptosis were found to be enhanced (Oikawa et al., 2022). We speculate that OTUD1 may be a key regulator of oxeiptosis.

Mitoquinone is a mitochondria-targeted antioxidant (Ismail et al., 2020). Several studies have verified that mitoquinone can cross the blood brain barrier and cellular membranes to accumulate in the mitochondria and lead to an increase in the expression of antioxidant enzymes (Ismail et al., 2020). In female granulosa cells and mouse oocytes, mitoquinone inhibits ROS-induced oxeiptosis by shifting energy metabolism. Mechanistically, mitoquinone effectively reduces the expression of AIFM1 and PGAM5 (Tsui and Li, 2023).

Breast cancer and ovarian cancer are common malignant tumors in women. Research has shown that many natural substances can inhibit the occurrence and development of these tumors (Ouyang et al., 2014). Alantolactone, an important sesquiterpene lactone, possesses antibacterial and anti-inflammatory properties (Liu et al., 2021). According to previous studies, alantolactone also has anti-cancer activity (Cai et al., 2021). Mechanistically, alantolactone promotes the accumulation of ROS by reducing glutathione and inhibiting thioredoxin reductase (Cai et al., 2021). Nasirzadeh M et al. observed that down-regulating NRF2 leads to decreased glutathione levels and increased ROS production (Nasirzadeh et al., 2023). In ovarian cancer cells, alantolactone modulates oxeiptosis by reducing NRF2 and boosting KEAP1 (Nasirzadeh et al., 2023). Alloimperatorin, a coumarin with anticancer properties (Bai et al., 2021), has been shown to significantly upregulate KEAP1 and decrease the phosphorylation of AIFM1 (Zhang et al., 2022). After downregulation of the KEAP1/PGAM5/AIFM1 expression, the inhibitory effect of alloimperatorin on cell viability was significantly reduced (Zhang et al., 2022). In breast cancer cells, alloimperatorin significantly inhibited cell invasion and growth by inducing oxeiptosis (Zhang et al., 2022).

Sanguinarine, a benzophenanthridine alkaloid derived from Sanguinaria canadensis (Croaker et al., 2017), has demonstrated antibacterial, anti-inflammatory, and antifungal properties (Huang et al., 2024). In studies both *in vitro* and *in vivo*, Pallichankandy S et al. found that sanguinarine suppresses the growth of human colorectal cancer cells by inducing oxeiptosis (Pallichankandy et al., 2023). On the one hand, the knockdown of KEAP1/PGAM5/IFM1 abolishes sanguinarine-induced oxeiptosis. Furthermore, sanguinarine triggered the dephosphorylation of AIFM1 at Ser116 in HT-29 cells and effectively inhibited tumor growth in the HT-29 xenograft mouse model through oxeiptosis. These results indicate that sanguinarine induces oxeiptosis by activating the KEAP1/PGAM5/AIFM1 signaling pathway.

Interestingly, nanoplastics (a polymer material) and TDCIPP (a building material) have also been shown to mediate oxeiptosis in both *in vitro* and *in vivo* studies (Guo et al., 2023; Wang et al., 2023). (Regulators of oxeiptosis in various diseases are shown in Table 1).

**TABLE 1:** Regulators of oxeiptosis in various diseases.

Regulator	Experimental model	Function	Ref.
circFOXO3	Human degenerated NP samples Male C57Bl/6 mice	Intervertebral disc degeneration ↑	Chen et al. (2024)
4-octylitaconate	The skin samples of psoriasis HacaT cells	Psoriasis ↓	You et al. (2023)
Phenethyl isothiocyanate	HepG2 and Hepa 1–6 cells	Hepatocellular carcinoma ↓	Strusi et al. (2023)
Dasatinib	HepG2 and Hepa 1–6 cells	Hepatocellular carcinoma ↓	Strusi et al. (2023)
OTUD1	HeLa and HEK293T cells Otud1^−/−^mice	Kidney cancer ↓	Oikawa et al. (2022)
Mitoquinone	Female granulosa cells and mouse oocytes	Stem cell proliferation and differentiation ↑	Tsui and Li (2023)
Alantolactone	Human ovarian cancer cell line (SKOV3)	Ovarian cancer ↓	Nasirzadeh et al. (2023)
Alloimperatorin	MCF-10A cells, MDA-MB-231 and MCF-7 human breast cancer cell lines	Breast cancer ↓	Zhang et al. (2022)
Sanguinarine	Human CRC cell lines (CaCo-2, HCT-116, and HT-115)	Colorectal cancer ↓	Pallichankandy et al. (2023)
Nanoplastics	Male C57BL/6 mice	Haematotoxicity ↑	Guo et al. (2023)
TDCIPP	Mouse testicular supporting cell line (TM4 cells)	Toxicity ↑	Wang et al. (2023)

## 6 Discussion

As research deepens, oxeiptosis has been identified in a variety of diseases. Additionally, oxeiptosis can play a significant role in conjunction with other cell death pathways. This combined approach may help address drug resistance in certain conditions. Therefore, understanding the molecular mechanisms of oxeiptosis is crucial. However, the study of oxeiptosis is still nascent, and many questions remain unanswered. Some studies have shown that AIFM1 can move into the nucleus and induce chromatin condensation (Hong et al., 2004). Yet, in oxeiptosis, AIFM1 remains within the mitochondria. Are there regulatory pathways for oxeiptosis beyond the KEAP1/PGAM5/AIFM1 pathway? How can foundational research on oxeiptosis be translated into clinical treatments? These are pressing issues that need resolution. In the future, we believe that the development of new oxeiptosis regulators has good prospects for treating and preventing related diseases. It is very meaningful to screen suitable oxeiptosis regulators from viruses, chemical drugs, natural substances, mRNA and polymer materials. Meanwhile, it is also valuable to explore whether other non-drug treatments such as acupuncture and diet are associated with oxeiptosis. In summary, the field of oxeiptosis is still in its early stages. Exploring ways to inhibit oxeiptosis and preserve more cells may become a critical research focus in the future.
